# Communicative interactions in point-light displays: Choosing among multiple response alternatives

**DOI:** 10.3758/s13428-015-0669-x

**Published:** 2015-10-20

**Authors:** Valeria Manera, Tabea von der Lühe, Leonhard Schilbach, Karl Verfaillie, Cristina Becchio

**Affiliations:** 1 CoBTek Laboratory, University of Nice Sophia Antipolis, Nice, France; 2 Department of Psychiatry and Psychotherapy, Heinrich-Heine-University of Düsseldorf, Rhineland State Clinics Düsseldorf, Düsseldorf, Germany; 3 Max Planck Institute of Psychiatry, Munich, Germany; 4 Department of Psychiatry, University Hospital Cologne, Cologne, Germany; 5 Laboratory of Experimental Psychology, KU Leuven, Leuven, Belgium; 6 Department of Robotics, Brain and Cognitive Sciences, Fondazione Istituto Italiano di Tecnologia, Genova, Italy; 7 Department of Psychology, University of Turin, Via Po 14, 10123 Turin, Italy

**Keywords:** Communicative interaction, Point-light, Biological motion, 5AFC, Database

## Abstract

Vision scientists are increasingly relying on the point-light technique as a way to investigate the perception of human motion. Unfortunately, the lack of standardized stimulus sets has so far limited the use of this technique for studying social interaction. Here, we describe a new tool to study the interaction between two agents starting from point-light displays: the Communicative Interaction Database - 5AFC format (CID-5). The CID-5 consists of 14 communicative and seven non-communicative individual actions performed by two agents. Stimuli were constructed by combining motion capture techniques and 3-D animation software to provide precise control over the computer-generated actions. For each action stimulus, we provide coordinate files and movie files depicting the action as seen from four different perspectives. Furthermore, the archive contains a text file with a list of five alternative action descriptions to construct forced-choice paradigms. In order to validate the CID-5 format, we provide normative data collected to assess action identification within a 5AFC tasks. The CID-5 archive is freely downloadable from http://bsb-lab.org/research/ and from the supplementary materials of this article.

**Electronic supplementary material:**

The online version of this article (doi:10.3758/s13428-015-0669-x) contains supplementary material, which is available to authorized users.

## Introduction

For humans, like many other species, survival depends on the ability to perceive what others are doing and predict what they may be intending to do. Biological motion provides a rich source of information in support of this skill (Blake & Shiffrar, 2007; Johansson, 1973). Human observers have no trouble identifying what an actor is doing in a given point-light display (e.g., Dittrich, 1993; Vanrie & Verfaillie, 2004). Even when the range of potential activities is quite large, they readily recognize individual actions and the associated emotions (Alaerts, Nackaerts, Meyns, Swinnen, & Wenderoth, 2011; Brownlow, Dixon, Egbert, & Radcliffe, 1997; Dittrich, Troscianko, Lea, & Morgan, 1996; Pollick, Paterson, Bruderlin, & Sanford, 2001; van Boxtel & Lu, 2011; Walk & Homan, 1984), are able to understand the intentions of the actor, and can detect a violation of his/her expectations (Runeson & Frykholm, 1983).

These findings highlight the importance of biological motion in the recognition of individual actions, i.e., actions performed by a single agent in isolation. Whether and how humans use biological motion to understand social interactions, however, is far less clear. In an influential study, Neri and colleagues (Neri, Luu, & Levi, 2006) first demonstrated that human observers integrate biological motion information from multiple individuals. Participants observed point-light displays of two agents fighting or dancing together. When the agents interacted in a meaningful synchronized fashion, visual detection of one agent was enhanced by the presence of the second agent. This suggests that the human visual system relies on the interaction dynamics between the two agents to retrieve information relating to each agent individually (see also Thurman & Lu, 2014). Subsequent studies extended these findings by showing that, even without any physical contact between the agents, the gestures of one agent can serve as a predictor of the actions of the second agent (Manera, Becchio, Schouten, Bara, & Verfaillie, 2011a; Manera, Del Giudice, Bara, Verfaillie, & Becchio, 2011b; Manera, Schouten, Verfaillie, & Becchio, 2013). Recent works have begun to explore perception of social interaction from biological motion in infants (Galazka, Roché, Nystrom, & Falck-Ytter, 2014) and pathological populations such as patients with autism spectrum disorder (Centelles, Assaiante, Etchegoyhen, Bouvard, & Schmitz, 2013; von der Luhe et al., submitted) and schizophrenia (Okruszek et al., 2015). The exact characteristics and the neural substrate supporting interpersonal action coding, however, remain unclear.

Although the point-light technique offers many advantages to researchers investigating perception of biological motion, the complexity of constructing stimuli depicting interacting point-light agents has so far limited the use of this technique in studying social interactions. Indeed, while several databases exist for the study of individual actions from point-light displays (Vanrie & Verfaillie, 2004; Ma, Paterson, & Pollick, 2006; Shipley & Brumberg, 2005), only few stimulus sets are available for the study of the actions of interacting agents (Manera et al., 2010; Zaini et al., 2013). Zaini and colleagues (Zaini, White, Fawcett, & Newman, 2013) recently published a database of point-light communicative hand gestures and non-communicative pantomimed hand actions. To the best of our knowledge, however, the only database to present two interacting agents, rather than just one, is the Communicative Interaction Database (CID; Manera, Schouten, Becchio, Bara, & Verfaillie, 2010).

The CID database contains 20 full-body communicative action sequences performed by two female and two male actors. Following Dekeyser, Verfaillie, and Vanrie (2002), stimuli were constructed by combining motion capture techniques and 3-D animation software to provide precise control over the computer-generated actions and allow the actions of the two agents to be independently manipulated.

In the present work, we describe the Communicative Interaction Database-5AFC format (CID-5), a new and updated version of the CID which addressed some limitations of the original CID (see Discussion section). The CID-5 consists of 14 communicative interactions selected from the CID – the best recognized stimuli based on the normative data (Manera et al., 2010) – and seven non-communicative individual actions performed by two agents, not included in the CID. For each action stimulus, we provide coordinate files and movie files depicting the action as seen from four different perspectives. Furthermore, for each action stimulus, we provide five action alternatives, including the correct action description, two communicative and two non-communicative individual alternatives to be employed in forced-choice response paradigms. In the following paragraphs we first describe the method used to construct the action stimuli and generate the response alternatives included in the 5AFC task, then we provide a detailed description of CID-5 database, including all the materials available for download. Finally, we provide normative data collected to assess the recognizability of the stimuli using the 5AFC format, and compare these results with those collected with an open-ended response format by Manera et al. (2010).

## Stimulus construction

A detailed and extensive discussion of the technical method has been published previously (Dekeyser et al., 2002; Vanrie & Verfaillie, 2004; Manera et al., 2010), so we limit the description of the stimulus construction to a summary of the major steps in the process.

### Motion capturing

The movements of four actors, two females and two males, each wearing 30 reflective spherical markers (placed on anatomical locations specified in Vicon’s Body-Builder 3.5 Manual; Oxford Metrics, 1997) were recorded using a Qualisys MacReflex motion capture system (Qualisys; Gothenburg, Sweden), consisting of six 30-Hz position units (i.e., six cameras and corresponding video processors). We recorded 14 communicative interactions and seven individual actions. For the communicative interactions, the two female and the two male actors worked in pairs and were assigned to a “communicator” and “responder” role. The communicator (agent A) always initiated the interaction by performing a communicative gesture; the responder (agent B) perceived the communicative gesture and acted in response. To ensure that the responder’s action matched the communicator’s gesture in all respects (e.g., timing, position, kinematics), interactions were captured in real time, with the actors facing each other, at a distance of approximately 2 m. Individual actions were performed by agent A acting in isolation. Objects (e.g., table, chair, coins, fruits) were present during the production of actions to aid the actors in producing natural movements.

### Data processing

After the capture session, the 2-D data from all the position units were processed offline to calculate the 3-D coordinates of the markers. The data from the markers were imported in Character Studio (Autodesk, 1998), a software package that was created for use with 3D Studio MAX (Autodesk, 1997). This allowed us to animate a biped for each actor, consisting of a transparent skeleton and 13 bright dots attached to the center of the major joints (shoulders, elbows, wrists, hips, knees, and ankles) and the head. Next, 3-D coordinates for each of the 13 dots for all the frames constituting each actor’s action were extracted, and some manual smoothing was performed to avoid any remaining “jumpy” dot movements.

To create the actual movie files, the smoothed data were imported into 3D Studio as moving bright spheres, and all the frames of the action were rendered as .avi files from four different viewpoints. An orthographic projection was used, and there was no occlusion, so no explicit depth cues were available. To create the communicative action stimuli .avi files, data from the two actors of each couple were imported into the same 3D studio environment. To create the individual action stimuli .avi files, we imported into the 3D Studio environment the 3D coordinates for all the frames constituting the action of agent B (the “responder” in the communicative interaction; e.g., “sitting down”) together with the 3D coordinates for all frames constituting an individual, unrelated action performed by agent A (e.g., “drinking”). For each individual action stimulus, the individual action was chosen so as to match the duration of the original communicative action performed by the “communicator,” and was displayed according to the original timing. Additionally, the distance and position with respect to agent B were kept constant. Objects present in the scene during motion capturing were never visible in point-light displays.

**Fig. 1:**
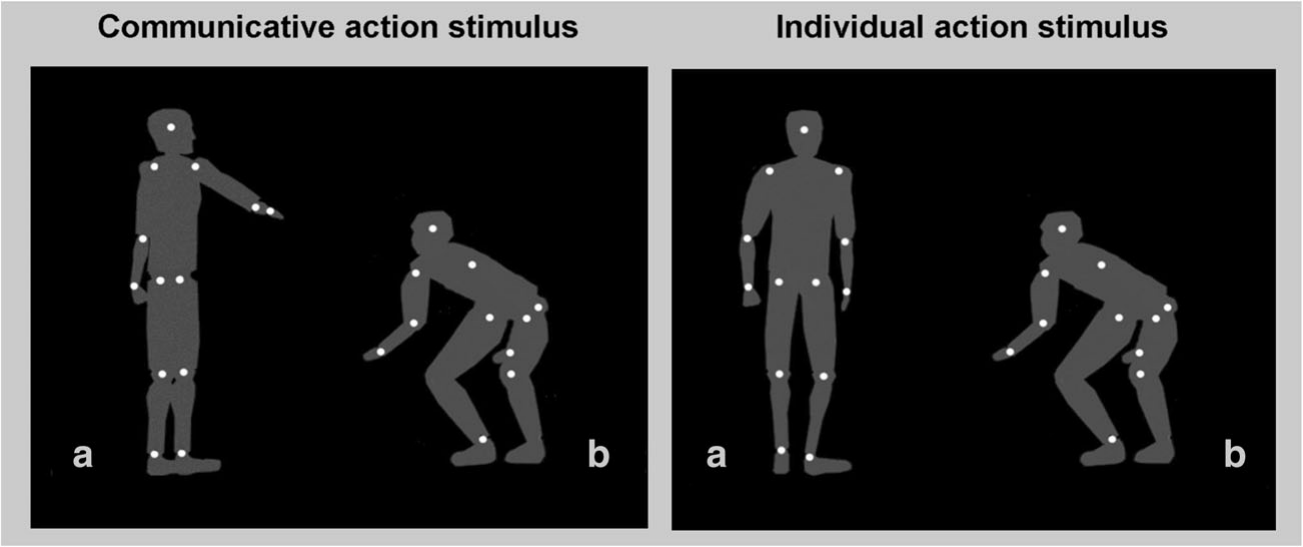
Example of a communicative action stimulus (agent A asks agent B to squat down) and an individual action stimulus (A turns over, B squats down). The grey silhouettes depicting the human form are not visible in the stimulus display

An example of a communicative and an individual action stimulus is reported in Fig. 1.

### Response alternatives

Based on the normative data collected by Manera et al. (2010), for each stimulus we selected the best recognized version, performed by either the male or the female couple. For each selected stimulus, we then generated five response alternatives, including the correct action description (based on the description provided to the actors) and four incorrect response alternatives, as reported in Table 2. The incorrect response alternatives were generated according to the following criteria. For each action stimulus (e.g., A asks B to walk away), two incorrect communicative alternatives (e.g., A opens the door for B; A asks B to move something) and two incorrect non-communicative alternatives (A stretches; A draws a line) were generated by modifying the description of the action of agent A. The descriptions provided by participants in the CID study (Manera et al., 2010) were used as a starting point, in order to ensure that the predetermined response alternatives included responses people would spontaneously give. All alternative action descriptions were constructed to be physically compatible with the action performed by agent A. For instance, if agent A performed an arm movement, then reference to arm movement was included in all incorrect response alternatives describing the action stimulus. Finally, to avoid that for communicative stimuli the correct alternative was selected simply based on the congruence between the actions of the two agents (i.e., agent A asks B to perform an action, and agent B responds *accordingly*), for each action stimulus, one of the incorrect communicative alternatives always described a congruent interaction between the two agents. The description of the action of agent B was the same for all response alternatives.

## The CID-5 Database

The database consists of a .rar archive that contains 21 folders, one folder for each of the actions listed in Table 1. Each action folder contains coordinate files and movie files depicting the action as seen from four different perspectives. Furthermore, the archive contains a text file with a list of the action alternatives provided for every action stimulus. The database can be retrieved from the supplementary materials of this article, or from the website of the Biology of Social Behavior Laboratory, University of Turin (http://bsb-lab.org/research/).

**Table 1:** Description and features of the action stimuli included in the CID-5. Please note that according to the taxonomy of speech acts (Searle, 1969, 1979), all the communicative (com) actions included in the CID-5 are classified as *directive*, meaning that the act of the communicator is intended to cause the responder to perform a certain action. Individual (Ind) action stimuli were not included in the CID

Action	Com vs. Ind	Action description	Gesture description	Duration, frames (ms)	Male / female couple	Object	Type of act	Social motivation	CID original name
**Choose which one**	Com	A asks B to choose between two objects. B takes an object	Holding two objects	265 (8833)	Male	Yes (apple and pear)	Directing	Offering	Which one
**Come closer**	Com	A asks B to come closer; B moves forward	Gesture of the hand	108 (3600)	Female	No	Directive	Giving instructions	Come closer
**Go out of the way**	Com	A asks B to go out of the way; moves over	Repeated movement of the hand	110 (3667)	Male	No	Directive	Giving instructions	Move over
**Imitate me**	Com	A squats down, and asks B to imitate him. B squats down	Pointing	313 (1043)	Male	No	Directive	Giving instructions	Imitate me
**Look at the ceiling**	Com	A asks B to look at something behind him on the ceiling. B turns around	Pointing	120 (4000)	Male	Yes (spider web)	Directive	Sharing	Look that on the ceiling
**Look at the ground**	Com	A asks B to look at something on the ground; B squats down	Pointing	134 (4467)	Male	Yes (flower)	Directive	Sharing	Look this on the floor
**Move this down**	Com	A asks B to move something down; B picks something and moves it down	Pointing	180 (6000)	Female	Yes (box)	Directive	Requesting	Put it down
**No**	Com	A says no; B, who had grasped something, puts that down	Gesture of the hand/ Arm	114 (3800)	Male	Yes (apple)	Directive	Ordering	No
**Pick this up**	Com	A points to B something to pick up. B picks something up	Pointing	138 (4600)	Female	Yes (coin)	Directive	Helping	Pick it up
**Sit down**	Com	A asks B to sit down; B sits down	Gesture of the arm/hand	130 (4333)	Male	Yes (chair)	Directive	Giving instructions	Sit down
**Squat down**	Com	A asks B to squat down; B squats down	Gesture of the hand	108 (3600)	Female	No	Directive	Ordering	Get down
**Stand up**	Com	A asks B to stand up; B, who is sitting, stands up	Gesture of the hand	100 (3333)	Female	Yes (chair)	Directive	Ordering	Stand up
**Stop**	Com	A asks B to stop; B, who is walking, stops.	Gesture of the hand	75 (2500)	Male	No	Directive	Ordering	Stop
**Walk away**	Com	A asks B to walk away. B takes some steps into the indicated direction	Indicating with the Hand	115 (3833)	Male	No	Directive	Ordering	Go over there
**Drink**	Ind	A drinks. B sits down	Lifting with the hand	130 (4333)	Male	Yes (glass and chair)	-	-	-
**Jump**	Ind	A jumps. B picks something up	Jumping up	138 (4600)	Female	Yes (coin)	-	-	-
**Lateral steps**	Ind	A makes some lateral steps. B takes something and eats it	Two lateral steps	245 (8167)	Male	Yes (apple)	-	-	-
**Look under the foot**	Ind	A looks under his foot. B moves something	Touching the foot	180 (6000)	Female	Yes (box)	-	-	-
**Sneeze**	Ind	A sneezes. B turns around	Hand movement	120 (4000)	Male	No	-	-	-
**Stretch**	Ind	A stretches. B moves something	Bending down to touch feet	151 (5033)	Female	Yes (flowerpot)	-	-	-
**Turn over**	Ind	A turns over. B squats down	Turning over 90°	108 (3600)	Female	No	-	-	-

### Actions

A brief description of each action stimulus is reported in Table 1. For each stimulus, we report the stimulus classification (communicative vs. individual), a brief description of the actions of agent A and agent B, the stimulus duration (number of frames and milliseconds), the actors’ gender, and the presence of (invisible) objects in the original scene. Furthermore, for the communicative stimuli, we also report the type of act (Searle, 1969, 1979) and the social motivation, representing why an agent performs a specific communicative gesture (e.g., sharing, requesting, asking for information; Tomasello, Carpenter, & Liszkowski, 2007), together with the original stimulus name in the CID database.

### Coordinate files

The coordinate files are listings of the 3-D coordinates of the point lights, saved in two different file formats: .txt and .pdf. Each action folder contains two coordinate files, representing the actions of the two actors. The filenames have the following structure: *action_role*, where *action* describes the specific action (communicative or individual) performed by agent A (listed in Table 1), and *role* is the role of the actor in the couple (A or B; in the communicative stimuli, A is the communicator performing the communicative gesture, B the responder acting in response).

The first line in the files provides the values of three parameters: the number of frames making up the action (dependent on the action), the number of markers (always 13), and the frame rate (always 30 Hz). The remainder of the file consists of the 3-D coordinates of the 13 markers for each frame of the action (Z-up coordinate system). The first 13 lines give the three coordinates of each of the 13 markers in the first frame, with the first number of each line indicating the width (x, oriented left to right), the second number indicating the depth (y, oriented towards the viewer), and the last number indicating the height (z, oriented bottom to top). The order of the point lights in the file is as follows: head, shoulders, elbows, wrists, hips, knees, and ankles. The subsequent 13 lines provide the coordinates for the second frame, and so on. The coupled actions of agent A and B always have the same number of frames. The 3-D coordinates are centered. That is, the averages of the horizontal, vertical, and depth coordinates of A and B coincide with the center of the coordinate system (0,0,0).

### Movie files

The movie files consist of point-light displays depicting the actions of the two agents in four different depth orientations: two lateral views (A positioned on the right [90°] and A positioned on the left [270°]), in which the coronal plane of the two actors is more or less perpendicular to the projection plane, and two three-quarter views (A on the right and seen from the front [125°] and A on the left seen from the back [305°]; see Manera et al., 2010 for further details). The filenames have the following structure: *action_ orientation*, where *action* describes the specific (communicative or individual) performed by agent A (listed in Table 1), and *orientation* describes the perspective (90°, 125°, 270°, and 305°). The two point-light figures, white against a black background, are approximately equidistant from the center of the screen. Movie files are .avi files with a resolution of 640 × 480 pixels and a frame rate of 30 frames/s.

### List of the response alternatives

The ‘Alternatives.doc’ file is a text file reporting the list of the five response alternatives for each action stimulus (see also Table 2).

**Table 2:** Response alternatives and results collected from 95 participants (percentage of participants who responded correctly to question 1, and who reported each of the action alternatives)

Action	Alternative	Alternatives	Com vs. Ind % (n=95)	Action % (n=95)
Choose which one	**Correct (COM)**	**A asks B to choose between two objects. B takes an object**	**100**	**76**
	COM -1	A offers something to B. B takes an object		11
	COM -2	A squats down and asks B to imitate him. B takes an object		2
	IND -1	A lifts something. B takes an object		4
	IND-2	A weights something in his hands. B takes an object		7
Come closer	**Correct (COM)**	**A asks B to come closer. B moves forward**	**98**	**82**
	_COM -1_	_A shows something to B. B moves forward_		7
	COM -2	A waves to B. B moves forward		8
	IND -1	A drinks. B moves forward		1
	IND-2	A stretches. B moves forward		1
Go out of the way	**Correct (COM)**	**A asks B to go out of the way. B moves over**	**97**	**26**
	_COM -1_	_A asks B to come closer. B moves over_		62
	COM -2	A asks B to hand him something. B moves over		3
	IND -1	A scratches himself. B moves over		4
	IND-2	A cleans something. B moves over		4
	COM -1 NEW	***NEW***. *A say hello to B. B moves over*		
Imitate me	**Correct (COM)**	**A squats down, and asks B to imitate him. B squats down**	**98**	**99**
	COM -1	A gives something to B. B squats down		0
	COM -2	A asks B to come closer. B squats down		0
	IND -1	A sits down. B squats down		1
	IND-2	A puts something down. B squats down		0
Look at the ceiling	**Correct (COM)**	**A asks B to look at something behind him on the ceiling. B turns around**	**86**	**73**
	COM -1	A asks B to turn. B turns around		19
	COM -2	A asks B to come closer. B turns around		0
	IND -1	A drinks. B turns around		2
	IND-2	A puts something down. B turns around		6
Look at the ground	**Correct (COM)**	**A asks B to look at something on the ground. B squats down**	**87**	**87**
	COM -1	A squats down and asks B to imitate him. B squats down		1
	COM -2	A asks B to move away. B squats down		1
	IND -1	A sits down. B squats down		3
	IND-2	A picks something up. B squats down		8
Move this down	**Correct (COM)**	**A asks B to move something. B moves something**	**65**	**62**
	COM -1	A asks B to imitate him. B moves something		0
	COM -2	A asks B to squat down. B moves something		4
	IND -1	A pours something. B moves something		24
	IND-2	A moves something. B moves something		9
No	**Correct (COM)**	**A says ‘No’. B stops**	**100**	**94**
	COM -1	A says ‘Hi’. B stops		2
	COM -2	A asks B to get out of the way. B stops		0
	IND -1	A rubs something. B stops		3
	IND-2	A shakes something. B stops		1
Pick this up	**Correct (COM)**	**A points to B something to pick up. B picks something up**	**84**	**83**
	COM -1	A asks B to squat down. B picks something up		1
	COM -2	A asks B to stop. B picks something up		1
	IND -1	A moves something. B picks something up		8
	IND-2	A checks the time. B picks something up		6
Sit down	**Correct (COM)**	**A asks B to sit down. B sits down**	**82**	**59**
	COM -1	A asks B to calm down. B sits down		22
	COM -2	A gives something to B. B sits down		1
	IND -1	A puts something down. B sits down		14
	IND-2	A folds something. B sits down		4
	COM -1 NEW	***NEW*** *. A asks B to move over. B sits down*		
Squat down	**Correct (COM)**	**A asks B to squat down. B squats down**	**97**	**76**
	COM -1	A asks B to pick something up. B squats down		3
	COM -2	A asks B to walk away. B squats down		0
	IND -1	A cleans something. B squats down		2
	IND-2	A bounces a ball. B squats down		19
Stand up	**Correct (COM)**	**A asks B to stand up. B stands up**	**85**	**79**
	COM -1	A shows something to B. B stands up		1
	COM -2	A asks B to squat down. B stands up		0
	IND -1	A bounces a ball. B stands up		13
	IND-2	A paints something. B stands up		7
Stop	**Correct (COM)**	**A asks B to stop. B stops**	**98**	**97**
	COM -1	A shows something to B. B stops		1
	COM -2	A asks B to sit down. B stops		0
	IND -1	A drinks. B stops		0
	IND-2	A puts something down. B stops		2
Walk away	**Correct (COM)**	**A asks B to walk away. B takes some steps**	**85**	**77**
	COM -1	A opens the door for B. B takes some steps		16
	COM -2	A asks B to move something. B takes some steps		1
	IND -1	A stretches. B takes some steps		4
	IND-2	A draws a line. B takes some steps		2
Drink	**Correct (IND)**	**A drinks. B sits down**	**92**	**78**
	COM -1	A asks B to sit down. B sits down		3
	COM -2	A asks B to look at something. B sits down		6
	IND -1	A looks at the time. B sits down		4
	IND-2	A scratches his head. B sits down		8
Jump	**Correct (IND)**	**A jumps. B picks something up**	**99**	**99**
	COM -1	A tells B he is very happy. B picks something up		0
	COM -2	A asks B to pick something up. B picks something up		1
	IND -1	A moves over. B picks something up		0
	IND-2	A lifts something. B picks something up		0
Lateral steps	**Correct (IND)**	**A makes some lateral steps. B takes something and eats it**	**88**	**68**
	COM -1	A offers something to B. B takes something and tastes it		6
	COM -2	A asks B to move over. B takes something and eats it		3
	IND -1	A turns around. B takes something and eats it		16
	IND-2	A pushes something. B takes something and eats it		6
Look under the foot	**Correct (IND)**	**A looks under his foot. B moves something**	**96**	**48**
	COM -1	A asks B to move something. B moves something		0
	COM -2	A asks B to look at something. B moves something		0
	IND -1	A stretches. B moves something		52
	IND-2	A turns around. B moves something		0
	IND -1 NEW	***NEW*** *. A kicks something. B moves something*		
Sneeze	**Correct (IND)**	**A sneezes. B turns around**	**82**	**76**
	COM -1	A asks B to look at something. B turns around		7
	COM -2	A asks B to sit down. B turns around		2
	IND -1	A eats something. B turns around		1
	IND-2	A throws something on the ground. B turns around		14
Stretch	**Correct (IND)**	**A stretches. B moves something**	**89**	**80**
	COM -1	A asks B to move something. B moves something		1
	COM -2	A asks B to squat down. B moves something		0
	IND -1	A picks something up. B moves something		14
	IND-2	A moves something. B moves something		5
Turn over	**Correct (IND)**	**A turns over. B squats down**	**100**	**91**
	COM -1	A shows something to B. squats down		0
	COM -2	A asks B to move away. B squats down		0
	IND -1	A moves something. B squats down		5
	IND-2	A moves away. B squats down		4

## Collection of normative data

In order to validate the 5AFC format of the CID-5 Database, we examined how well each stimulus was recognized by naïve participants and then compared these results with those collected by Manera et al. (2010) on CID stimuli employing an open response format.

### Participants

One hundred and thirteen students from the Faculty of Psychology at the University of Turin (Italy) volunteered to take part in this study, and received course credits for their participation. Eighteen participants were excluded from data analysis due to missing responses, leaving the final sample to 95 participants (ten of them male and 85 female; mean age= 22.9 years, SD= 5.6, age range= 19–58). All the participants were naive as to the purpose of the study and had no previous experience with point-light displays.

## Methods

Participants were tested in group in a conference room (250 seats arranged in ten rows), with a central projection screen. The 21 action stimuli were presented in a randomized order. Each action stimulus consisted of the same action repeated from two different perspectives (90° and 125°), with the two videos separated by a 500-ms fixation cross. After the second repetition of each video, participants were asked to decide whether the two agents were communicating as opposed to acting independently of each other (question 1), and then to select the correct action description among the five response alternatives, presented in a randomized order (question 2).

## Results

For each question and for each stimulus, we calculated whether the proportion of correct responses differed from chance level – that is, from .5 for question 1 (corresponding to 50 % of correct responses) and .2 for question 2 (corresponding to 20 % of correct responses) –by employing binomial tests. Bonferroni corrections were applied to adjust for multiple comparisons (α = .05/21, = .0023).

The percentage of participants who responded correctly to each action stimulus is reported in Table 2. The “Com vs. Ind” column indicates the percentage of correct responses to question 1 (classification of the action as communicative vs. individual). The column “Action” indicates the percentage of responses provided for each of the five response alternatives. The first action alternative (in bold) reports the correct description.

### Classification as communicative versus individual

On average, action stimuli were correctly classified as communicative versus individual (question 1) by 91 % of the participants (SD= 8 %; range= 65–100 %; communicative stimuli, M= 90 %, SD= 10 %; individual stimuli, M= 92 %, SD= 6 %). The action that was least consistently recognized was “Move this down” (correctly classified as communicative by 65 % of the participants). Classification of the action as communicative versus individual was above chance level for 20 out of the 21 action stimuli (all ps ≤ .001). ). For the action “Move this down,” the binomial test just approached statistical significance (p = .004).

### Action identification

Concerning the selection of the correct action description (question 2), on average, action stimuli were correctly identified by 77 % of the participants (SD= 17 %; range= 26–99 %). Examples of very well recognized stimuli are “Stop” (97 %) and “Imitate me” (99 %) for the communicative action stimuli, and “Jump” (99 %) and “Turn over” (91 %) for the individual action stimuli. Although the overall recognition rate was high, some consistent misidentification occurred. For example, for “Go out of the way,” the incorrect action description “A asks B to come closer. B moves over” was selected by 62 % of the participants. Similarly, 52 % of the participants misidentified “Look under the foot” as “A stretches. B moves something.” Binomial tests revealed that participants performed significantly better than the chance level for 20 out of 21 action stimuli (all ps < .001). Performance was not above the chance level only for the action “Go out of the way” (p = .084).

For action stimuli identified by less than 60 % of the participants and for which more than 20 % of the participants selected the same incorrect action description (n =3; “Go out of the way,” “Sit down,” and “Look under the foot”), in Table 2 we provide an alternative action description to be used as a substitute for the confusable incorrect description. These new action descriptions were employed in another study (Manera et al., submitted), and were only rarely (or never) selected by healthy adults, thus suggesting that they were indeed less misleading compared to the alternatives tested in the present study. [Please refer to Manera et al. (submitted) for more details about results and procedures.]

## Comparison between the CID and the CID-5 databases

The main differences in the materials included in the CID database and the CID-5 database are summarized in Table 3.

**Table 3:** Summary of the materials provided in the CID and CID-5

	CID	CID-5
No. of communicative actions	20	14
No. of individual actions	-	7
No. of couples performing each action	2	1
No. of movie files for each action	4	4
Coordinates files for each actor/action	Yes	Yes
Response alternatives for each actor/action	No	Yes
No. of participants for the normative data	54	95

In order to investigate differences in action identification between the forced-choice paradigm employed in the CID-5 database and the open-ended response format employed in the CID database (where participants were asked to generate a description of each action stimulus, see Manera et al., 2010), action identification accuracy for each action stimulus was submitted to separate chi-square tests, with Group (CID vs. CID-5) as the between-subjects factor. A Bonferroni correction was applied to adjust for multiple comparisons (α = .05/21, = .0023). The results revealed no significant difference between the open response format and the forced-choice format for 19 out of 21 action stimuli (χ^2^ ranging from .00 to 6.61, φ ranging from − .21 to .19, all ps > .011). A significant difference was found for the actions “Go out of the way” (χ^2^ = 53.94, φ = −.60, p < .001) and “Look under the foot” (χ^2^ = 13.94, φ = −.31, p < .001). Specifically, the percentage of correct responses for those actions was significantly lower for the CID-5 forced-choice format (26 % for “Go out of the way,” 48 % for “Look under the foot”) compared to the CID open-ended response format (89 % for “Go out of the way”, 80 % for “Look under the foot”), thus confirming that some response alternatives in the 5AFC task were very misleading.

Taken together, these results indicate that the 5AFC employed in the CID-5 and the open response format employed in the CID (Manera et al., 2010) yield comparable levels of accuracy in action identification for most of the action stimuli.

## Discussion

In the present paper we describe the CID-5, a database of 21 full-body point-light stimuli depicting two agents engaged in communicative interactions (N=14) or performing non-communicative individual actions (N=7) as seen from different viewpoints. For each stimulus, we provided five plausible response alternatives (only one being correct), and we collected normative data on 95 naive participants to assess stimulus recognizability. Results confirm that stimuli included in the CID-5 are highly recognizable, thus suggesting that information contained in these point-light displays is sufficient to distinguish communicative interactions from non-communicative individual actions, and to recognize the specific communicative or individual actions performed by the agents.

We are convinced that our stimulus set may represent a useful tool for researchers working on communication and social cognition, since an important factor in the advancement of those studies is the availability of suitable stimulus material. In particular, the CID-5 has several advantages compared to the existing databases of communicative actions, such as the CID (Manera et al., 2010). First of all, the CID-5 contains communicative interactions and non-communicative individual actions directly comparable in terms of the method used for stimulus construction, stimulus format (size, distance between the actors, available viewpoints), and action duration. The presence of individual control stimuli is crucial to test whether human observers are able to discriminate between communicative and non-communicative action stimuli, i.e., to test whether they are sensitive to interaction dynamics (Manera et al., 2011a; b; 2013). Furthermore, the presence of well-matched control stimuli represents a key advantage for studies aiming to investigate the neural correlates of social interaction (Centelles et al., 2011).

Second, in the CID-5 we provided for each action stimulus five possible response alternatives (the correct action description, two incorrect communicative alternatives, and two incorrect non-communicative alternatives) to create a forced-choice response paradigm. Asking participants to provide a free description of the stimuli can capture the spontaneous intention attribution process and is sometimes the best option. However, use of open-ended responses may be problematic in certain experimental setups and populations. For instance, open-ended responses may be difficult to answer for children with developmental expressive language disorder (Simms & Schum, 2011), patients with acquired brain damage (Angeleri et al., 2008), patients with neurodegenerative pathologies such as Alzheimer’s disease (Henry, Crawford, & Phillips, 2004), and patients with autism and schizophrenia (Groen, Zwiers, Vandergaag, & Buitelaar, 2008; Delisi & Lynn, 2001). An additional advantage of the forced-choice questions is that they decrease the number of missing responses, as the participant can be prompted to select one of the alternatives. Furthermore, the forced-choice format can facilitate response scoring, a process that can sometimes be challenging with the open-response format, especially when employing visually degraded stimuli (such as point-light animations) representing complex human actions.

Third, we reported normative data collected on a large sample of participants (N=95), specifying not only the percentage of correct responses for each action stimulus, but also the percentage of participants that selected each of the incorrect response alternatives. These data may be used as a guideline by researchers interested in creating easier or more challenging versions of the task. On the one hand, a 3AFC format may simplify the intention recognition task, especially if the more misleading alternatives are removed from the response list. Similarly, easier versions of the task may be obtained by randomizing the alternatives across different action stimuli, so that the proposed alternatives are physically incompatible with the displayed action. On the other hand, more challenging versions may be obtained by increasing the number of alternatives. A 7AFC task, for example, may be more challenging than the original 5AFC task, especially if the new alternatives are similar to the correct response or to the misleading alternatives.

## Limitations and future research directions

Despite the important advantages of the CID-5 compared to the existing databases of communicative actions, some limitations should be noted. First of all, the CID-5 contains a limited number of non-communicative individual stimuli (N=7). This may represent a problem for studies needing to employ a larger number of stimuli (e.g., neuroimaging studies). However, it should be noted that in the CID-5 we also provided listings of the 3-D coordinates of the point lights for each stimulus and for each actor. This allows researchers to create different non-communicative individual stimuli by combining different actions performed by agent B (the respondent in the communicative interactions).

Another limitation lies in the paradigm employed for the collection of normative data assessing stimulus recognizability. Specifically, participants were presented with the same action stimulus as seen from two different perspectives, a lateral view and a three-quarter view, and were asked to select the correct response alternative only after the second repetition of the video. However, we know from previous studies that the representations of both individual actions and social interactions are viewpoint-dependent (Daems & Verfaillie, 1999; De la Rosa, Mieskes, Bülthoff, & Curio, 2013; Verfaillie, 1993, 2000) and that viewpoint modulates the cortical response to visually presented actions (e.g., Kilner, Marchant, & Frith, 2006; Perrett et al., 1989). It would thus be important to collect normative data assessing separately the recognizability of each action perspective. These data may represent further guidelines to be employed for stimulus selection.

## Electronic supplementary material

Below is the link to the electronic supplementary material.

ESM 1 (RAR 21.3 mb)
